# Supplementary material for: R. verniciflua and E. ulmoides Extract (ILF-RE) Protects against Chronic CCl4-Induced Liver Damage by Enhancing Antioxidation
Source: Nutrients. 2019 Feb 12;11(2):382. doi: 10.3390/nu11020382 (PMC6412399; doi:10.3390/nu11020382)
Supplement: Supplementary file 1 [file nutrients-11-00382-s001.pdf]

Supplementary Materials and Method:

Quantitation of fustin, fisetin, and sulfuretin using HPLC analyses

The HPLC analysis of individual fustin, fisetin, and sulfuretin were performed on an HPLC system equipped with an Agilent 1260 Infinity II (Agilent Technologies, Santa Clara, CA, USA). Stock standard solutions of fustin, fisetin, and sulfuretin were prepared in 70% methanol at a concentration of 1.0 mg/mL. The appropriate amount of each standard solution was mixed and diluted with 70% methanol as indicated. Chromatographic separation was carried out using a Polaris C8 column (4.6 × 250 mm, 5 μm, Agilent Technologies, Wilmington, DE, USA) heated to 30 °C. The injection volume was 5 μL, and the following solvent ratios were used for the mobile phase with a flow rate (1 mL/min: where solvent A is : 0.1% formic acid in water and solvent B is 0.1% formic acid in acetonitrile): 0 min, 20% B; 10 min, 20% B; 30 min, 40% B. The detection wavelength (280 nm) was scanned at a flow rate (1 mL/min). Peak analyses and assignments were performed using fustin, fisetin, and sulfuretin, which were identified in accordance with their UV spectra and retention times in the HPLC chromatograms.

Quantitation of geniposide and geniposidic acid using HPLC analyses

The HPLC analysis of individual geniposide and geniposidic acid were performed on an HPLC system equipped with an Agilent 1260 Infinity II (Agilent Technologies, USA). Stock standard solutions of geniposide and geniposidic acid were prepared in 50% methanol at a concentration of 1.0 mg/mL. The appropriate amount of each standard solution was mixed and diluted with 50% methanol as indicated. Chromatographic separation was carried out using a SB-C18 column (4.6 × 150 mm, 3.5 μm, Agilent Technologies, Wilmington, DE, USA) heated to 25 °C. The injection volume was 5 μL, and the following solvent ratios were used for the mobile phase with a flow rate (1 mL/min: where solvent A is: 0.1% formic acid in water and solvent B is acetonitrile): 0 min, 5% B; 5 min, 5% B; 15 min, 15% B; 25 min, 15% B; 25.5 min, 90% B; 30 min, 90% B. The detection wavelength (254 nm) was scanned at a flow rate (1 mL/min). Peak analyses and assignments were performed using geniposide and geniposidic acid, which were identified in accordance with their UV spectra and retention times in the HPLC chromatograms.

Supplementary Figures:

A

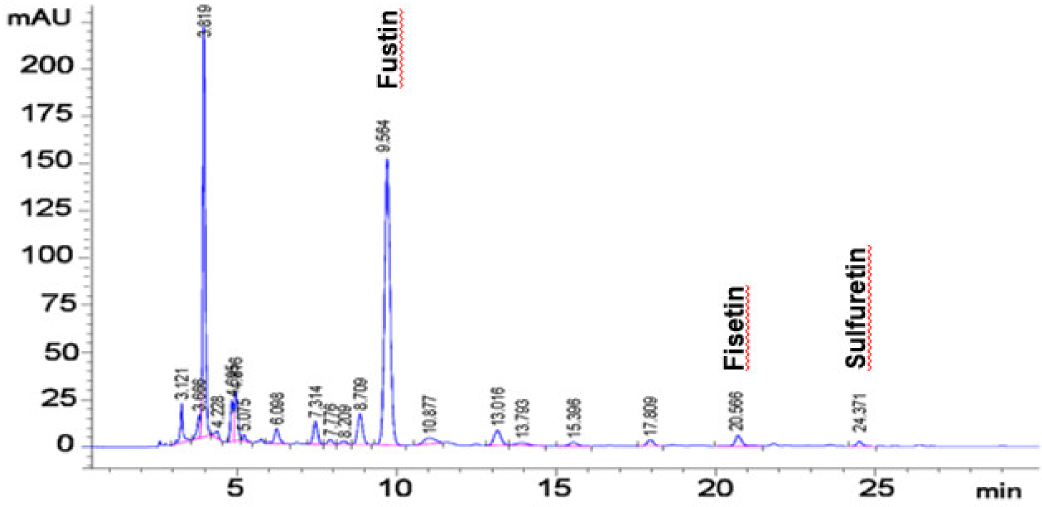

B

	Retention time (Time)	Area (mAU*s)	Amount (ng/uL)	Content (mg/g)
Fustin	9.564	1861.08813	106.43487	53.76
Fisetin	20.566	84.41827	19.60186	9.34
Sulfuretin	24.371	31.56856	3.92641	1.94

Figure S1. Full scan mass spectrum of fustin, fisetin, and sulfuretin. (**A**) HPLC chromatogram: ILF-R (*Rhus verniciflua)* extract; (**B**) Concentration of fustin, fisetin, and sulfuretin in *Rhus verniciflua* extracts.

A

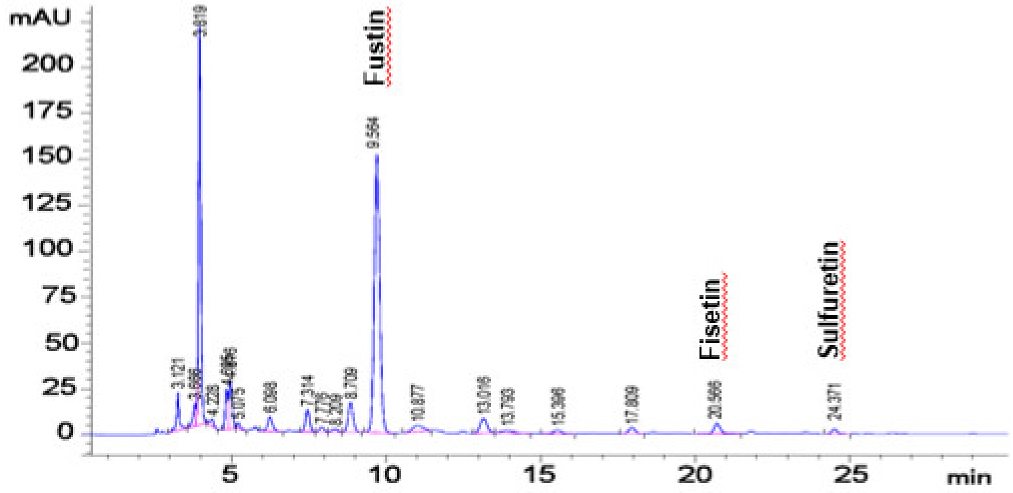

B

	Retention time (Time)	Area (mAU*s)	Amount (ng/uL)	Content (mg/g)
Fustin	9.564	1861.08813	106.43487	53.76
Fisetin	20.566	84.41827	19.60186	9.34
Sulfuretin	24.371	31.56856	3.92641	1.94

Figure S2. Full scan mass spectrum of geniposide and geniposidic acid. (**A**) HPLC chromatogram: ILF-E (*Eucommia ulmoides)* extract; (**B**) Concentration of geniposide and geniposidic acid in *Eucommia ulmoides* extracts.

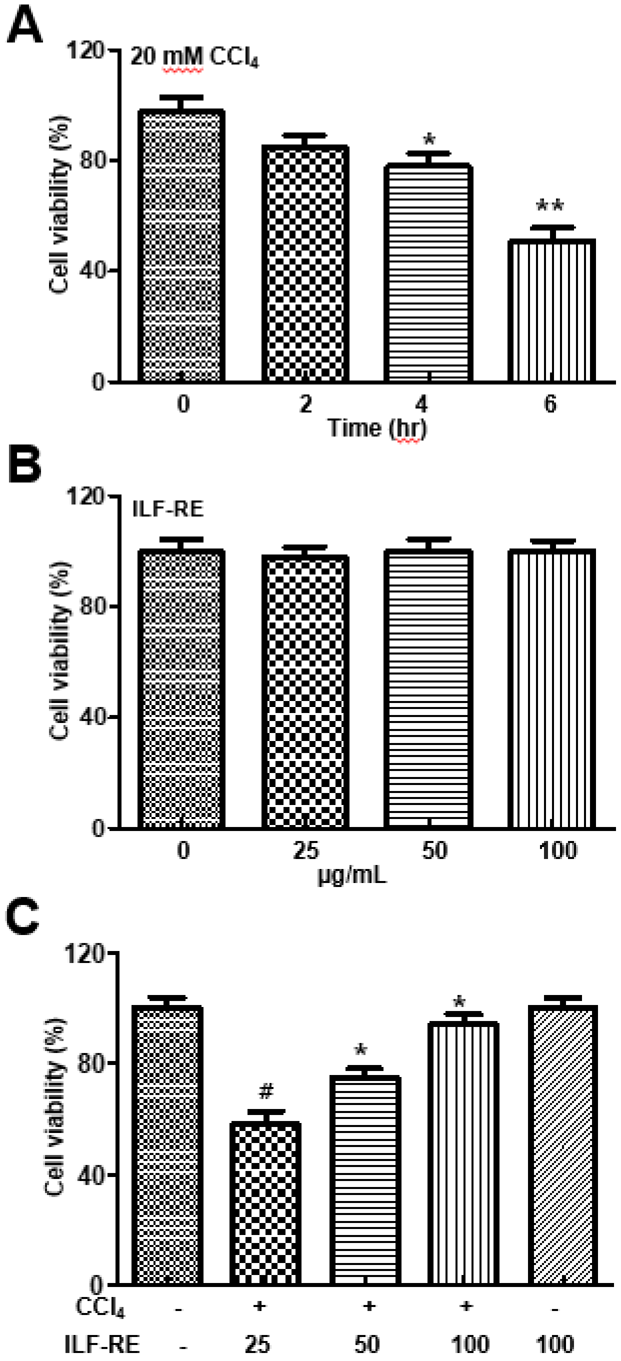

Figure S3. ILF-RE protects hepatocyte cells from CCl_4-induced toxicity. (**A**) Hepatocyte cells were incubated with 20 mM CCl_4 for 2, 4, or 6 h. Cell viability was assessed as described in the materials and methods section ($n = 3$, $^{\#}p < 0.05$ vs. Con, $^{*}p < 0.01$ vs. Con). (**B**) Hepatocyte cells were treated with 25, 50, or 100 μg/mL water extracts of ILF-RE for 6 h. Cell viability was assessed as described in the materials and methods section. (**C**) Hepatocyte cells were treated with 25, 50, or 100 μg/mL water extracts of ILF-RE, then incubated with 20 mM CCl_4 for 6 h Cell viability was assessed as described in the materials and methods section. Data are expressed relative to the controls ($n = 3$, $^{\#}p < 0.05$ vs. CCl_4, $^{*}p < 0.01$ vs. CCl_4). Con, control; CCl_4, carbon tetrachloride; ILF-RE, the combination *Rhus verniciflua* with *Eucommia ulmoides.*

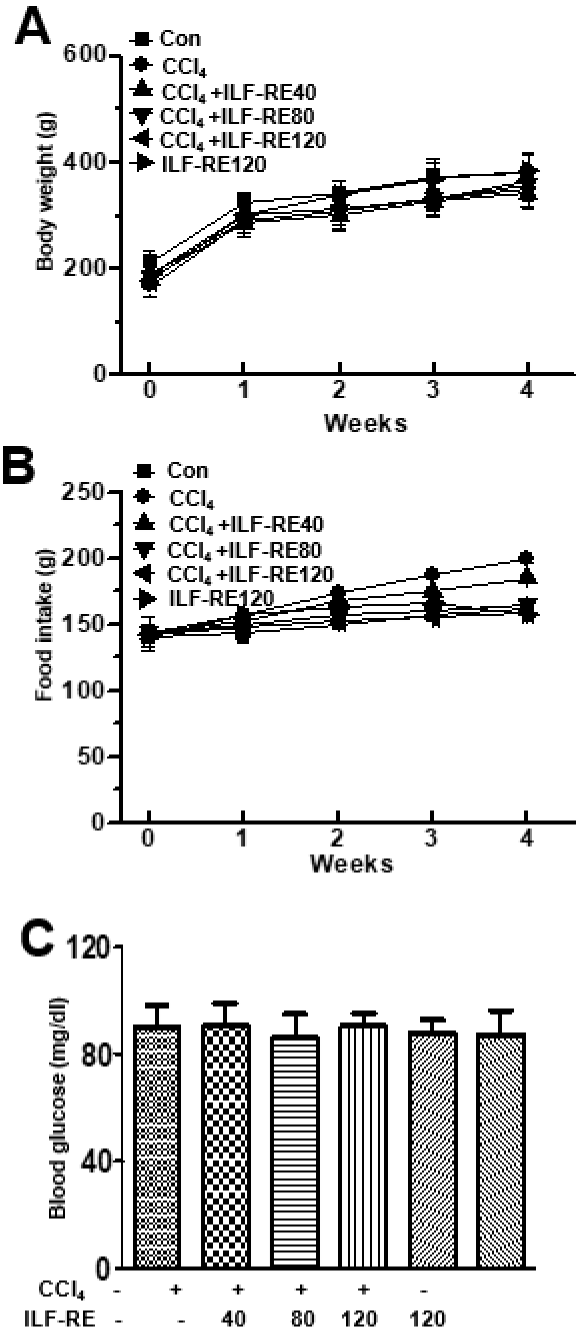

Figure S4. ILF-RE protects body weight, food intake, and blood glucose from CCl_4-induced toxicity. Rats were intraperitoneally treated with CCl_4 (0.2 mL/100 g, body weight) twice a day for 4 weeks. ILF-R (40, 80, or 120 mg/kg) was given with CCl_4 treatment or 120 mg/kg ILF-RE only for 4weeks. Body weight (**A**), food intake (**B**), and blood glucose (**C**) were measured. The experiments were repeated three times using tissues from at least three different rats. $^{\#}p < 0.05$ *versus* con group, $^{*}p < 0.05$ *versus* CCl_4 group (n = 8 rats per group). Con, control; CCl_4, carbon tetrachloride; ILF-RE, the combination *Rhus verniciflua* with *Eucommia ulmoides.*
